# The Intake of Omega-3 Fatty Acids, the Omega-3 Index in Pregnant Women, and Their Correlations with Gestational Length and Newborn Birth Weight

**DOI:** 10.3390/nu16132150

**Published:** 2024-07-05

**Authors:** Ksenija Nikolajeva, Olga Aizbalte, Roberta Rezgale, Vinita Cauce, Dzintars Zacs, Laila Meija

**Affiliations:** 1 Riga East Clinical University Hospital, 2 Hipokrata Street, LV-1038 Rīga, Latvia; laila.meija@rsu.lv; 2 Doctoral Department, Faculty of Medicine, Rīga Stradiņš University, 16 Dzirciema Street, LV-1007 Rīga, Latvia; 3 Faculty of Master’s Study Program, Nutrition Science, Riga Stradiņš University, 16 Dzirciema Street, LV-1007 Rīga, Latvia; 020999@rsu.edu.lv; 4 Faculty of Medicine, Rīga Stradiņš University, 16 Dzirciema Street, LV-1007 Rīga, Latvia; roberta.rezgale@gmail.com (R.R.); vinita.cauce@rsu.lv (V.C.); 5 Scientific Institute of Food Safety, Animal Health, and Environment, Lejupes Street 3, LV-1076 Rīga, Latvia; dzintars.zacs@bior.lv; 6 Department of Public Health and Epidemiology, Rīga Stradiņš University, 9 Kronvalda bulvāris, LV-1010 Rīga, Latvia

**Keywords:** pregnancy, nutrition, fatty acids, omega-3, omega-3 index, erythrocytes

## Abstract

Background: During pregnancy, the demand for omega-3 fatty acids, notably docosahexaenoic acid (DHA), escalates for both maternal and foetal health. Insufficient levels can lead to complications and can affect foetal development. This study investigated omega-3 status and its relation to dietary intake in pregnant Latvian women, along with its impact on gestational duration and newborn birth weight. Methods: The study comprised 250 pregnant and postpartum women with a mean age of 31.6 ± 4.8 years. Nutrition and omega-3 supplementation data were collected through a questionnaire covering 199 food items and 12 supplements. Fatty acids in erythrocyte membrane phospholipids were analysed via gas chromatography with flame ionization detection. Results: The median omega-3 fatty acid intake, including eicosapentaenoic acid (EPA) and DHA from diet and supplements, was 0.370 g/day, which is deemed sufficient. However, the median weekly fish intake (126.0 g) and daily nut/seed intake (7.4 g) were insufficient. The median omega-3 supplement intake was 1.0 g/day. No correlation between omega-3 supplement intake and the omega-3 index was observed. There was a weak correlation between the DHA intake from fish and the omega-3 index (r = 0.126, *p* = 0.047), while a significant correlation between the total EPA and DHA intake from various sources and the omega-3 index was noted (r = 0.163, *p* = 0.01). Most women (61.6%) had an omega-3 index < 4%, while 34.8% had an index between 4 and 8%, and only 3.6% had an index > 8%. Notably, significant differences in EPA levels and the omega-3 index were found among respondents with differing infant birth weights (*p* < 0.05). Conclusions: The omega-3 intake during pregnancy adheres to the established guidelines, although fish consumption remains insufficient. A preconception evaluation of the omega-3 index is advocated to optimize prenatal intake. The indications suggest potential correlations between EPA levels, the omega-3 index, and infant birth weight.

## 1. Introduction

Studies have highlighted the importance of omega-3 fatty acids during pregnancy. Eicosapentaenoic acid (EPA) and docosahexaenoic acid (DHA) are phospholipids that form the cell membrane (especially in the retina and brain) [1]. Fatty acids are transported from the pregnant woman to the foetus via the placenta. Fish is the primary food source of EPA and DHA. EPA and DHA are synthesized from the essential fatty acid alpha-linolenic acid (ALA) present in flaxseed oils and walnuts. However, the efficiency of ALA conversion in human adults is low (5% to EPA and <1% to DHA) [2].

Most authoritative bodies and expert scientific organizations recommend omega-3 intake during pregnancy and lactation in the range of 0.30 g (EPA + DHA) per day, of which DHA should be at least 0.20 g/day. The most common recommendation is to consume 2–3 weekly servings of fish, including fatty sea fish once a week [3,4]. The consumption of fish and shellfish that contain DHA and EPA is more efficient than ALA conversion in increasing omega-3 levels to meet this increased requirement. At the same time, there are some precautions that fish consumption exposes the foetus to methylmercury and other environmental contaminants.

The omega-3 index, defined as the percentage of EPA and DHA in 26 fatty acids measured in erythrocytes, represents the human tissue status of EPA and DHA. The omega-3 index has low biological variability and is a long-term parameter. Previous studies considered that the optimal range of the omega-3 index during pregnancy and lactation is 8–11% [5].

Evidence suggests that the intake of EPA and DHA has potential benefits such as an improvement in foetal neurodevelopment and reduced risks of pre-eclampsia and metabolic diseases, in addition to effects on gestational duration and birth weight [1,6].

Preterm birth, defined as delivery before 37 weeks of gestation, impacts approximately 10% of pregnancies worldwide, is a major cause of perinatal mortality and morbidity, and is often attributed to spontaneous labour onset or maternal and foetal complications [7,8]. Low plasma concentrations of EPA and DHA during pregnancy significantly elevate the risk of early preterm birth [8]. 

Low birthweight (LBW), defined as a birthweight below 2500 g irrespective of gestational age, accounts for over 80% of neonatal fatalities, while macrosomia, characterized by infants weighing over 4000 g, also poses significant risks of morbidity and long-term health challenges, including the onset of adult chronic conditions such as cardiovascular disease [9,10]. The clinical effects of omega-3 correlate better with its levels in the blood than with its intake [5]. Despite the primary focus of professional recommendations on dietary intake, the data suggest that if not supplemented, DHA levels in pregnant women will decrease in proportion to the amount transferred to the foetus [11]. Previous studies have indicated that the omega-3 index in pregnant women is insufficient [12].

This study focused on evaluating the omega-3 fatty acid index in pregnant women and its association with dietary intake from both food and supplements. Additionally, it aimed to investigate the adequacy of the recommended dietary omega-3 intake during pregnancy in achieving optimal omega-3 index values while also exploring correlations between erythrocyte omega-3 levels and gestational duration as well as newborn birth weight.

## 2. Materials and Methods

### 2.1. Study Population

This cross-sectional study was conducted under the Latvian Council of Science project “Excess weight, dietary habits, and vitamin D and omega-3 fatty acid status in pregnancy”, project No. Izp-2019/1-0335. Prior to the commencement of the project, quota sampling was employed to stratify the target population based on the proportion of women of reproductive age across different regions of Latvia. This stratification was based on demographic data from the Central Statistical Office as of early 2019, accounting for seasonal variations. According to the Central Statistical Office, there were 296,354 women of reproductive age in Latvia in 2019, which served as a reference for calculating the sample quota. The final sample comprised 800 postpartum women and 200 pregnant women. Our study comprises only a portion of the data. Data collection occurred from July 2020 to August 2021. The study sample consisted of 250 healthy pregnant women between the 27th and 40th weeks of gestation, as well as postpartum women up to 7 days after delivery. Birth outcomes were recorded for the postpartum participants. Data on nutritional status and supplements were collected during the last 6 months of pregnancy. Surveys were conducted among participants from ambulatory institutions in Riga and stationary maternity wards in various cities in Latvia. Exclusion criteria were age under 18 years, individuals with a history of diabetes, celiac disease, inflammatory bowel disease, short bowel syndrome, gastrointestinal tract, and eating disorders, as well as individuals residing outside Latvia. 

### 2.2. Data Collection

For the purposes of this study, the food frequency questionnaire was adapted from the one developed by the Scientific Institute of Food Safety, Animal Health, and Environment “BIOR” [13]. The questionnaire included 199 food products and beverages grouped into 20 product groups and 12 supplement positions. During the survey, the “Photo Atlas of Food Products and Food Portions” was used to clarify the portions of food products and beverages. The questionnaire on demographics, lifestyle, health status, and nutritional habits consisted of 73 questions and included information on various factors, including medical history, anthropometric data, demographic data, the use of nutritional supplements, lifestyle, and physical activity. Additionally, data from medical documentation, including anthropometric data, blood tests, pregnancy progression, and complications, were collected.

### 2.3. Cutoff Values 

According to the Nordic Nutrition Recommendations (2012) [14], European Food and Safety Authority [15], and Food and Agriculture Organization of the United Nations (FAO) [16] recommendations for pregnant and lactating women, the minimum daily intake of EPA + DHA is 0.30 g, of which DHA should be at least 0.20 g. The Dietary Guidelines for Americans 2020–2025 [3] recommend that pregnant or lactating women consume 225–340 g of a variety of seafood per week, which, on average, corresponds to 0.340–0.530 g [17] of DHA per day. The recommended daily intake of ALA is 30 g of nuts (15 g walnuts, 7.5 g hazelnuts, and 7.5 g almonds) [18]. Most reports have stated that omega-3 index values of ≥8% are optimal for pregnancy and lactation [19,20,21,22]. We used cutoff values of proposed risk zones of omega-3 index levels of <4%, 4–8%, and >8%, corresponding to deficit, intermediate, and optimal levels, respectively [20].

According to the World Health Organisation, preterm birth is defined as birth prior to 37 weeks of gestation, including extremely preterm (<28 weeks), very preterm (28–<32 weeks) and moderate or late preterm (32–<37 completed weeks of gestation) [23,24]. Low birthweight (LBW) was characterized by a birthweight below 2500 g, and foetal macrosomia as birth weight > 4000 g [9,25].

### 2.4. Blood Samples

Blood tests were performed for participants in certified laboratories immediately after the survey for postpartum women, up to 7 days after delivery, or 1 week after the survey for pregnant women. Blood samples were processed, frozen, and delivered to the Scientific Institute of Food Safety, Animal Health, and Environment “BIOR” for analysis within 13 days of collection. Pregnant women received the test results via email. Personal data were kept confidential, with further data processing using assigned identification codes.

### 2.5. Measurement of FA Composition in Erythrocytes

Fatty acids in the phospholipids of the erythrocyte membranes were determined at BIOR using gas chromatography coupled with a flame ionization detector (GC–FID).

The tube containing the blood sample was removed from the freezer and thawed. Then, it was mixed, and 200 µL was pipetted into another tube. Then, 800 µL of distilled water was added to the 200 µL erythrocyte sample and centrifuged for 10 min at 3000 rpm. The resulting mass containing phospholipid membranes was rinsed with 800 µL of distilled water and centrifuged again to obtain erythrocyte membranes. Then, 400 µL of distilled water and 3 mL of a chloroform–methanol solution (1:1 ratio) were added and mixed. The chloroform layer was transferred to another tube, and the solvent was removed by evaporation. Phospholipids were hydrolysed and methylated simultaneously with 100 µL of butylhydroxytoluene and 0.5 mL of a boron triphosphide/methyl alcohol mixture for 60 min at 100 °C in closed tubes in a heating block. After cooling, 800 µL of distilled water and 800 µL of hexane were added for fatty acid extraction. The supernatant (hexane) containing the fatty acids was transferred to a clean glass container, evaporated to dryness under nitrogen gas, and re-dissolved in 100 µL of hexane for GC–FID analysis. Fatty acid detection was conducted using GC model—Agilent 6890 N with an FID detector, and the sample set was processed in Agilent Chemstation (Supelco, Bellefonte, PA, USA). The results are expressed as the percentage of individual fatty acids relative to the total fatty acids.

### 2.6. Ethics

The Clinical Research Ethics Committee of Riga Stradiņš University granted ethical approval (No. 6-1/02/62) for the study. Before the survey, participants received written information about the study’s objectives and purposes. After providing written consent, the participants were given a code for data processing to encode the data obtained.

### 2.7. Data Analysis

Dietary data from the dietary frequency questionnaire were analysed at BIOR using the Institute’s custom programme based on Microsoft Dynamics Ax 2009. This programme utilises the BIOR Food Composition database, initially created to analyse food consumption data from the “Food Consumption Study of the Latvian Population (2012–2013)”, and it continues to provide data for other Latvian population food consumption studies. The “BIOR” food composition database is based on the food composition database created by the German Max Rubner Institute and includes Latvian products and recipes. An analysis of dietary data provided information on the consumption of total energy, nutrients, individual fatty acids, and vitamins.

Qualitative data were represented using numbers and percentages, while quantitative data were described using either the mean and standard deviation or the median and interquartile range (Q1–Q3), depending on the data distribution. The chi-squared test was employed to determine the association between the omega-3 index and nutrient intake. The Mann–Whitney test was utilized to compare the means of erythrocyte lipid parameters during pregnancy based on gestation length, and the Kruskal–Wallis H test with Bonferroni correction was applied to compare the means of erythrocyte lipid parameters during pregnancy based on infant birth weight. Statistical significance was set at *p* < 0.05. Statistical analyses were conducted using SPSS version 27.0.

## 3. Results

### 3.1. Baseline Characteristics 

Table 1 shows the baseline characteristics of the study participants. The study included 182 (72.8%) postpartum women and 68 (27.2%) pregnant women. The average participant of the study was a married Latvian woman with a mean age ± SD of 31.6 ± 4.8 years, with higher education and a normal body mass index (BMI). Nevertheless, 31.8% (*n* = 78) of the respondents had overnutrition (overweight or obese) before pregnancy.

Information regarding the weight of newborns can be found in Table 2. The majority of infants of the respondents fell within the normal birth weight category, but both low birth weight (LBW) and large for gestational age (LGA) infants were also observed.

The exercise and smoking status data are summarised in Table 3. Women who walked more than 30 min a day made up 80.4% (*n* = 202) of the sample, and 34.4% (*n* = 86) performed physical exercise at least 2–3 times a week, but more than half of all the participants (64%, *n* = 160) engaged in sports only sometimes or never.

### 3.2. Nutrients and Omega-3 Fatty Acid Intake

Table 4 shows participants’ energy, nutrient, and omega-3 fatty acid intakes. The fat intake during pregnancy was 40.3%, of which the proportion of saturated fat was 39.1%. The daily median intake of EPA and DHA was 140 (58–287) mg and 220 (116–427) mg, respectively.

Fatty acids were consumed from different food groups. Table 5 shows the dietary intake of omega-3 fatty acids during the study period.

Table 6 shows the amounts of omega-3 foods consumed by the respondents. Fish was consumed by 91.6% (*n* = 229) of all respondents, where the median weekly amount was 126.0 (55.2–265.8) g. Nuts and seeds were consumed by 92.0% (*n* = 230) of respondents in a median amount of 7.4 (2.8–18.4) g daily. However, omega-3 supplements were consumed by 35.2% (*n* = 80) in the amount of 1.0 g/day.

### 3.3. Omega-3 Index and Correlation with the Intake of Omega-3 Sources and Lifestyle

Most respondents (61.6%, *n* = 154) had an omega-3 deficiency (omega-3 index < 4%), 34.8% (*n* = 87) of respondents had an omega-3 index of 4%–8%, while only 3.6% (*n* = 9) had an omega-3 index of >8%.

Table 7 shows the consumption patterns of omega-3 sources and the omega-3 index. From the sample of respondents who ingested fish, nuts and seeds, and omega-3 supplements during pregnancy, 5.1% (*n* = 4) had an omega-3 index of at least 8%. Conversely, for respondents who did not ingest fish, nuts and seeds, or omega-3 supplements, only 2.9% (*n* = 5) of that sample had an omega-3 index of at least 8. However, no significant correlation was found between the omega-3 index and the consumption of omega-3 sources (*p* > 0.05).

For women who consumed fish at least 300 g/week (*n* = 49, 19.6%), the median level of the omega-3 indexes did not correspond to the optimal level of the omega-3 index (3.5 [3.0–4.0]). Additionally, women who consumed more than 0.2 mg of DHA per day (*n* = 139, 55.6%) also had an omega-3 index lower than 4 (3.5 [3.0–3.9]). No correlation was found between omega-3 supplement intake and omega-3 index (*n* = 88, *p* > 0.05). Nevertheless, there was a weak correlation between the total intake of DHA from fish and the omega-3 index (r = 0.126, *p* = 0.047, *n* = 250) and a significant correlation between the total intake of EPA and DHA from different omega-3 sources (fish, nuts and seeds, fats, and omega-3 supplements) and the omega-3 index (r = 0.163, *p* = 0.01, and *n* = 250). 

No significant correlation was found between the omega-3 index and time of delivery, smoking habits, BMI, or physical exercise schedule. 

Table 8 presents a summary of the omega-3 fatty acids, the omega-3 index, and pregnancy outcomes. Although no statistically significant correlation was observed between the lipid parameters and pregnancy duration, a statistically significant difference was identified in the EPA level and the omega-3 index among respondents with varying infant birth weights.

## 4. Discussion

During pregnancy, it is vital to maintain a healthy weight, pursue an appropriate amount of physical activity, and provide the body with the necessary nutrients for a successful pregnancy and foetal development. 

An evaluation of the participants’ BMI before pregnancy showed that 64.4% of the entire sample had a normal BMI, but 31.8% were overweight or obese. Omega-3 fatty acids have a protective effect against cardiometabolic risks, which can also arise due to obesity. A high BMI may be associated with a low omega-3 index. However, the results showed no correlation between BMI and the omega-3 index. Possible explanations are that there were no women with severe obesity in our study and the potential effect of pregnancy on women’s metabolism and omega-3 status. 

The proportional distribution of nutrients consumed by the participants did not correspond with the recommendations for a healthy diet for pregnant women. Although the average amount of energy consumed was within the recommended limits, the distribution of nutrients did not correspond to the recommended distribution. On average, this study’s participants consumed a lot of fat (>40%), primarily saturated fat; such a proportion did not contribute to higher omega-3 intake and its bioavailability at appropriate levels. These results are consistent with the data from a systematic review of 18 studies on the nutritional habits of pregnant women from 10 different countries worldwide. In these studies, it was observed that pregnant women in other countries also had a low consumption of vegetables and cereal products, as well as an excessive intake of fat (>30% of the total energy intake) [26,27]. Additionally, their diets did not meet the nutritional recommendations developed by the WHO for pregnancy, which poses an increased risk of adverse perinatal outcomes.

The omega-3 index was insufficient in most participants, reflecting omega-3 deficiency in tissues, whereas only a minor proportion of participants (3.6%) had an omega-3 index of >8%.

Our study participants consumed an average of 360 mg of EPA and DHA through their diet and omega-3 supplements, of which 220 mg was DHA. In a cross-sectional study in the USA that enrolled 1260 pregnant women, the mean daily intake of EPA and DHA was 78.7 mg and 88.1 mg through food and omega-3 supplements, respectively [28]. Another study from Germany found that pregnant women’s mean daily intake of DHA was 100 mg/day [12]. According to recommendations for pregnant women, the daily DHA intake in our study was considered appropriate. However, the findings indicated that the dietary intake of fish among Latvian women during pregnancy did not meet the recommended levels. Specifically, the median daily consumption of DHA derived from fish was 178 mg, while the average weekly fish intake was 126 g. According to the Dietary Guidelines for Americans 2020–2025, pregnant and lactating women should consume 225–340 g of a variety of seafood per week or at least 200 mg of DHA per day. German women consumed 441–588 g of fish per week during pregnancy, which is more than the recommended amount of fish intake. The lower fish intake by pregnant Latvian women may be explained by caution regarding fish consumption due to the risk of contamination with heavy metals, thus reducing the intake of omega-3 fatty acids, especially DHA.

The results showed a weak positive correlation between the omega-3 index and diet, especially the amount of omega-3 fatty acids ingested from fish, and a significant correlation between the total intake of EPA and DHA from different omega-3 sources (fish, nuts and seeds, fats, and omega-3 supplements) and the omega-3 index. This suggests that foods containing omega-3 fatty acids, especially fish, have high bioavailability in the human body. However, pregnant women who consumed more than 300 g of fish per week (*n* = 49) or >200 mg of DHA per day (*n* = 139) did not show an optimal omega-3 status (3.5 [3.0–4.0]). As there was a weak correlation between the total DHA intake from omega-3 sources and the omega-3 status, the current tests should be performed with a larger sample size.

Because of the comprehensive metabolic pathways of ALA conversion to DHA, and even though in women, especially during pregnancy, the body’s ability to convert ALA into EPA and, accordingly, into DHA increases, the conversion process depends on many factors such as genetic factors, endogenous fatty acid metabolism, certain health conditions, and others. These factors were not analysed as confounding factors in this study, and there is no strong recommendation regarding the amount of ALA intake for pregnant and lactating women. However, the participants of this study did not reach the recommended amount of 30 g/day of nuts and seeds.

Considering the possible neurotoxicity of heavy metals contained in fish and the negative impact on the cognitive development of the foetus, as well as taking into account the fact that the dietary habits of some pregnant women do not include fish, it is recommended to compensate for the intake of omega-3 fatty acids EPA and DHA using omega-3 supplements, which indicates that pregnant women who did not consume omega-3 fatty acids in the diet (for example, through fish) compensated for the lack of omega-3 fatty acids in the body by taking nutritional supplements. According to the data from a German study, only 11.5% of pregnant women consumed additional omega-3 fatty acid supplements [12], three times lower than the results of our study conducted in Latvia. However, considering that no positive correlation was found between the intake of omega-3 fatty acids from supplements and the omega-3 index, it is suggested that the mean intake of omega-3 was too low to detect a positive correlation. Our research collected and evaluated data on the intake of omega-3 sources and supplements in the last 6 months of pregnancy and compared it with the omega-3 index, a long-term indicator of omega-3 status in tissues. However, information on how regularly each pregnant woman took the respective supplement, whether a doctor prescribed it, and whether it was taken following the recommendations and instructions for the supplement was not available. Based on the data collected in a German cross-sectional study, pregnant women who consumed the recommended amount of omega-3 fatty acid supplements had a positive correlation between blood omega-3 levels and the amount of fatty acids consumed. However, the omega-3 index still did not reach the desired 8–11% range [12,29], which could indicate that the recommended dose during pregnancy was insufficient. =This could be explained by the fact that, as the requirements for omega-3 fatty acids increase during pregnancy, especially in the third trimester, when DHA accumulates in the foetal brain membranes and subcutaneous tissue for future development, the current recommendations for the daily intake of 200 mg DHA are insufficient during pregnancy.

It is imperative to acknowledge that the outcomes observed in this study may have been impacted by the varying omega-3 content present in wild and cultured fish. It is pertinent to acknowledge that wild fish typically exhibit higher levels of *n*-3 fatty acids compared to their farmed counterparts. This distinction arises primarily due to the dietary differences between the two groups, with oceanic and marine fish primarily feeding on phytoplankton and zooplankton rich in *n*-3 fatty acids, while farmed fish often consume vegetable oils, which are abundant in *n*-6 polyunsaturated fatty acids [30,31]. Furthermore, it is essential to consider that dietary supplements encompass various forms of omega-3, including natural triglycerides, free fatty acids, and ethyl esters, etc. [32]. The bioavailability of omega-3 fatty acids can significantly differ depending on their chemical binding, i.e., lipid structure [30,31], thereby potentially influencing the observed outcomes. One additional limitation of this study is the lack of assessment of storage-induced oxidant stress. This phenomenon can induce a cascade of biochemical and morphological changes, potentially impacting membrane lipids by promoting the release of fatty acids from complex membrane structures, which could serve as a mechanism for damage repair [33].

Other factors such as physical activity, smoking habits, and BMI did not show a relationship or influence on the omega-3 index in the correlation tests. Additionally, no association was observed in our study between the omega-3 fatty acid levels and gestational length. A systematic review and meta-analysis revealed that the supplementation of omega-3 long-chain polyunsaturated fatty acids (LCPUFAs) in pregnant women was associated with a 35% reduction in early preterm births (<34 weeks’ gestation) and a 12% reduction in preterm births (<37 weeks’ gestation), along with a modest increase in mean gestational length [34]. However, another systematic review involving 3854 women did not find a reduction in preterm births with omega-3 supplementation [35]. Some research findings suggest the presence of a potential correlation between maternal DHA levels and preterm birth among mothers exhibiting the lowest DHA levels [36,37]. Given the potential inflammatory effects and other complications associated with insufficient omega-3 levels in preterm births, we suggest that future research with larger sample sizes is needed. Our result showed that the EPA and omega-3 index levels were different between LBW, LGA, and normal weight infants. Data from a meta-analysis suggested that omega-3 LCPUFAs were associated with a decreased likelihood of infants being born with a low birth weight (LBW), and there was a potential modest rise in the risk of infants being born large for gestational age (LGA) [1]. According to the research data, omega-3 LCPUFA intake demonstrates potential in mitigating risks associated with a low birth weight while necessitating further exploration in clinical studies [6,34,35,36,37]. 

In the future, considering that no correlations were found between the intake of fish and omega-3 supplements in recommended amounts and the omega-3 index, it is necessary to study these variables in larger samples to determine omega-3 fatty acid recommendations through diet or supplements to contribute to an increase in the omega-3 index in pregnant women, especially during the third trimester of pregnancy.

## 5. Conclusions

Based on this study, fish consumption by Latvian women during pregnancy is insufficient. Although the recommended total amount of omega-3, including in food and supplementation, was appropriate, it did not lead to advisable omega-3 index values. There are suggestions that lipid parameters, EPA, and the omega-3 index in erythrocytes may exhibit associations with infant birth weight. However, further research in the Latvian population is needed to confirm these associations. 

We recommend reviewing the recommendations of nutrition and nutritional supplements during pregnancy, determining the omega-3 index when planning a pregnancy, and adjusting the omega-3 intake, especially in the third trimester of pregnancy when the need for omega-3 in the body is at its highest.

## Figures and Tables

**Table 1:** Participants’ demographic characteristics (*n* = 250).

Characteristic	*n* (%)
Participant	
Pregnant women	68 (27.2)
Postpartum women	182 (72.8)
Length of gestation < 37 weeks	11 (6.1)
Length of gestation ≥ 38 weeks	170 (93.9)
Nationality	
Latvian	179 (71.6)
Russian	55 (22.0)
Other	16 (6.4)
Marital status	
Married	169 (67.6)
Live in partnership	78 (31.3)
Divorced	1 (0.4)
Single mother	1 (0.4)
Education	
Primary/incomplete secondary education	13 (5.2)
General special/secondary education	43 (17.3)
College education	7 (2.8)
Higher education	170 (68.6)
Incomplete higher education	15 (6.1)
BMI before pregnancy	
<18.5	7 (2.8)
18.5–24.95	161 (65.4)
25.0–29.9	54 (22.0)
≥30.0	24 (9.8)

BMI—body mass index, kg/m^2^.

**Table 2:** Characteristic of newborn weight (*n* = 182).

Newborn Weight, g	*n* (%)
<2500	8 (4.4)
2500–4000	138 (75.8)
>4000	36 (19.8)

**Table 3:** Lifestyle characteristics during pregnancy (*n* = 250).

Variable	*n* (%)
Time spent walking and cycling per day	
<15 min	9 (3.6)
15–30 min	37 (14.9)
30–60 min	108 (43.5)
>60 min	94 (37.9)
Frequency of at least 30 min of physical exercise	
I cannot exercise due to illness or disability	4 (1.6)
Sometimes or never	110 (44.7)
2–3 times a month	19 (7.6)
Once a week	27 (11.0)
2–3 times a week	62 (25.2)
4–6 times a week	12 (4.9)
Every day	12 (4.9)
Physical activity at work	
I do not work	20 (8.0)
Easy	102 (41.0)
Medium	74 (29.7)
Heavy handwork	52 (20.9)
Smoking during pregnancy	
Yes	18 (7.3)
No	229 (92.7)

**Table 4:** Daily intake of nutrients from food and supplements (*n* = 250).

Energy/Nutrient/Fatty Acids	Median (Q1–Q3)
Energy (kcal)	2229 (1753–2804)
Protein (g)	102.2 (80.3–129.4)
Carbohydrate (g)	216.8 (162.3–269.5)
Fibre (g)	22.3 (16.1–29.4)
Fat (g)	99.9 (77.3–131.2)
SFA (g)	39.1 (27.8–50.0)
MUFA (g)	38.9 (28.8–47.7)
PUFA (g)	17.2 (11.6–21.6)
Omega-3 FA	
ALA (g)	2.10 (1.59–2.95)
EPA (g)	0.14 (0.06–0.29)
DHA (g)	0.23 (0.12–0.43)

SFA—saturated fatty acid; MUFA—monounsaturated fatty acid; PUFA—polyunsaturated fatty acid; FA—fatty acid; ALA—α-linolenic acid; EPA—eicosapentaenoic acid; DHA—docosahexaenoic acid. Dietary intakes were estimated from a dietary frequency questionnaire.

**Table 5:** Median omega-3 fatty acid intake (g/day) from main food sources and supplements (*n* = 250).

Food Groups	ALA (Q1–Q3)	EPA (Q1–Q3)	DHA (Q1–Q3)
Nuts and seeds	0.39 (0.02–0.48)	n/a	n/a
Fats *	0.47 (0.09–0.57)	n/a	n/a
Fish	0.03 (0.05–0.38)	0.11 (0.18–0.15)	0.18 (0.02–0.24)
Soups	0.07 (0.03–0.10)	0.01 (0.00–0.01)	0.00 (0.00–0.00)
Omega-3 supplements	n/a	0.10 (0.00–0.10)	0.06 (0.00–0.07)

* Olive, flaxseed, and hemp oils. Dietary intakes were estimated from a dietary frequency questionnaire. ALA—α-linolenic acid; EPA—eicosapentaenoic acid; DHA—docosahexaenoic acid; n/a—not available.

**Table 6:** Omega-3-rich food intake, g/day.

Omega-3 Sources	*n* (%)	Median Intake (Q1–Q3)
Fish (per week)	229 (91.6)	126.0 (55.2–265.8)
Nuts and seeds	230 (92.0)	7.35 (2.8–18.4)
Walnuts	167 (66.8)	0.98 (0.24–2.46)
Peanuts	92 (36.8)	3.05 (0.24–2.46)
Peanut butter	72 (28.8)	1.64 (0.33–4.80)
Almonds	176 (70.4)	2.46 (0.43–6.41)
Chia seeds	98 (39.2)	0.32 (0.08–2.13)
Pumpkin seeds	100 (40)	0.32 (0.08–0.82)
Flaxseeds	90 (36)	0.32 (0.08–2.13)
Hemp seeds	26 (10.4)	0.32 (0.08–2.13)
Flaxseed oil	37 (14.8)	2.49 (0.09–2.56)
Omega-3 supplements	88 (35.2)	1.00 (0.49–2.50)

Dietary intakes were estimated from a dietary frequency questionnaire.

**Table 7:** Omega-3 index and consumption of omega-3 sources ^a^.

Product	Omega-3 Index			*p* Value
	Deficit, <4%	Intermediate, 4–8%	Optimal, >8%	
Fish				
Consume	142 (62.0)	79 (34.5)	8 (3.5)	0.891
Do not consume	12 (57.1)	8 (38.1)	1 (4.8)	
Nuts/seeds				
Consume	143 (62.2)	78 (33.9)	9 (3.9)	0.452
Do not consume	11 (55.0)	9 (45.0)	0 (0.0)	
Fish and nuts/seeds				
Consume	134 (63.2)	70 (33.0)	8 (3.8)	0.373
Do not consume	20 (52.6)	17 (44.7)	1 (2.6)	
Omega-3 supplements				
Consume	54 (61.4)	30 (34.1)	4 (4.5)	0.700
Do not consume	100 (61.7)	57 (35.2)	5 (3.1)	

^a^ Chi-squared test was used to analyse associations between variables. Dietary intakes were estimated from a dietary frequency questionnaire.

**Table 8:** Erythrocyte lipid parameters in pregnancy depending on length of gestation and birth infant weight.

Lipid Parameters	Length of Gestation Median (Q1–Q3)		*p* Value ^a^	Infant Weight Median (Q1–Q3)			*p* Value ^b^
	<37 Weeks	≥38 Weeks		<2500	2500–4000	>4000	
ALA	0.50 (0.30–0.60)	0.50 (0.40–0.70)	0.410	0.55 (0.30–0.75)	0.50 (0.40–0.70)	0.40 (0.40–0.65)	0.460
EPA	1.90 (1.50–2.20)	2.00 (1.70–2.40)	0.337	2.00 (1.65–2.35)	1.90 (1.60–2.40)	2.15 (1.95–2.70)	0.016 *
DHA	3.60 (3.35–3.85)	3.40 (2.70–4.00)	0.304	3.55 (3.40–4.05)	3.30 (2.70–4.10)	3.40 (2.55–3.85)	0.355
Omega-3 index	3.30 (3.10–3.75)	3.40 (2.90–3.70)	0.927	3.45 (3.20–4.15)	3.30 (2.80–3.70)	3.50 (3.30–3.90)	0.012 *

^a^ Mann–Whitney U test used to compare means between groups. ^b^ Kruskal–Wallis H test with Bonferroni correction was used to compare means between groups. * *p* < 0.05. ALA—α-linolenic acid; EPA—eicosapentaenoic acid; DHA—docosahexaenoic acid.

## Data Availability

The data presented in this study are available upon request from the corresponding author.
